# How can we improve the diversity of archival collections with AI? Opportunities, risks, and solutions

**DOI:** 10.1007/s00146-025-02222-z

**Published:** 2025-02-24

**Authors:** Lise Jaillant, Olivia Mitchell, Eric Ewoh-Opu, Maribel Hidalgo Urbaneja

**Affiliations:** 1 Loughborough University, Loughborough, UK; 2 Royal College of Art, London, UK; 3 University of the Arts London, London, UK

**Keywords:** Archives, Diversity, Artificial intelligence, Ethics

## Abstract

This article is the first study to examine the impact (positive and negative) of Artificial Intelligence on the diversity of archival collections. Representing the diverse audiences they serve is a key objective for libraries and archives. For example, institutions with colonial-era archival documents are experimenting with AI to improve the discoverability of their collections and to enhance access for source communities and other users. Indeed, AI can be used to automatically create metadata, search vast amounts of historical records, and answer questions with natural language. However, these technologies also come with risks—for instance when AI systems are trained on potentially biased data. Very little is known about the impact of these computational tools on diversity in archival collections. Do AI technologies compound or alleviate the lack of diversity in archives? Drawing from interviews with academics, archivists, curators, and other experts across the UK/Europe and the USA, this article sheds light on the lack of collaboration between producers of AI technologies on the one side, and archivists, librarians and other cultural heritage professionals on the other side. We argue that bringing these stakeholders together is essential to improve the diversity of archival collections, using ethical and responsible AI. Finally, we offer recommendations to help professionals in libraries and archives assess the opportunities and risks associated with AI and find solutions to make their collections more representative of diverse audiences.

## Introduction

This article explores how AI technologies can help address the critical question of lack of diversity in archival collections. The Oxford English Dictionary defines “diversity” as “the fact, condition, or practice of including or involving people from a range of different social and ethnic backgrounds, and (more recently) of different genders, sexual orientations, etc.”^1^ This meaning of the term originates in the cultural revolution of the 1960s, at a time of contestation of established powers. Rooted in a specific historical moment, this push for more gender, sexual, and racial diversity has impacted numerous academic disciplines—including archival studies—and professional practice. There is a widespread consensus that archival collections should reflect the diverse audiences served by cultural heritage institutions and be easily accessible to them.

Equality, diversity and inclusion (EDI) frameworks and policies are widely used to ensure ethical and fair treatment of all social groups, and in particular the ones that are commonly subject to discrimination on the basis of their identity. While this article focuses mainly on archives and libraries, it is important to note that the broader GLAM^2^ sector is increasingly incorporating EDI statements in institutional codes of practice. For instance, the UK Science Museum Group developed an Equity Framework, which underscores the need to be mindful of “who is represented and whose ideas and cultures are centred and valued” in their collections. This framework outlines the museum’s approach to tackling lack of diversity as aiming to “identify exclusion and inequity,” “foster an equitable environment,” and “reflect and embed structural change.”^3^ In the USA, the Art Institute of Chicago published a statement on Equity, pledging to “advanc[e] racial justice now and in the future.” The statement recognises that the institution’s main site on Michigan Avenue, which opened in 1893, is located on lands once inhabited by Indigenous people. The list of iconic artists, carved into the building’s neoclassical entablature, focuses on White, Western European, male artists. “The omission of artists of colour, especially Black artists, as well as female, Indigenous, and non-Western artists, is glaring.”^4^ Such examples are reflective of a general acknowledgement of the issue of lack of diversity in the GLAM sector.

The common understanding of the term “diversity” has changed over time. Racial diversity became increasingly prominent in the wake of the Black Lives Matter movement and the global protests in 2020 following the murder of George Floyd. In contrast, other categories—such as class—have moved down in importance. For Gabriele Griffin (Professor Emerita of Gender Research, Uppsala University), “gender is no longer as high on the agenda as it once was.”^5^ Others have argued (and sometimes deplored) that what’s on the decline is not gender per se, but “the narrow, heterosexual, white and Eurocentric performances of womanhood with which many feminists had mostly concerned themselves to date” (Stock 2020).

In the first stages of this research, we left the definition of “diversity” broadly open, but we soon realised that the professionals we interviewed referred mostly to race and ethnicity. This has oriented the focus of this article, which examines the lack of diversity in archives, understood in two ways: (1) the underrepresentation of archival collections documenting racial and ethnic communities and/ or (2) the difficulties for these racialised audiences to access archival materials (for example, in the case of sensitive colonial archives). The origins of these issues are multifaceted, stemming from historical biases as well as structural and systemic issues such as socio-economic disparities. They are also context specific: “lack of diversity” will be understood differently in the USA, which has a problematic history of slavery and marginalisation of Black Americans, and in the UK/ Europe where colonisation shaped multiple archival collections.

Many scholars have examined the question of diversity in archival collections. In the context of analogue collections, Harrison et al. (2020) have explored “how ‘diversity’ emerged historically as a normative conservation target across a range of different forms of natural and cultural heritage preservational practices throughout the course of the twentieth century.” Some have focused on diversity from a theoretical viewpoint in archival studies (Caswell 2021) or in the context of colonial archives (Burton 2005; Stoler 2008). Others have adopted a more practical approach applied to specific collections—such as web archives (Bingham and Byrne 2021). Diversity is thus a key priority for the collection-as-data initiative, which draws on the Global Indigenous Data Alliance’s CARE Principles (Carroll et al 2020) and work on Maori Data Sovereignty.^6^

In Australia, North America and the UK, new protocols have been proposed to address the issue of racist and outdated language in archival collections. The overall aim of these protocols is to "decolonise archival descriptive practices" (Chilcott 2019). Projects such as "Provisional Semantics" (funded by the Arts and Humanities Research Council in the UK) have focused on redressing outdated language, and on interrogating how problematic descriptions are produced and reinforced in catalogue entries and object descriptions (Pringle et al 2022).

Moving the focus towards technological tools and solutions, other academics and practitioners have looked at the use of Artificial Intelligence in archives (Colavizza et al. 2021; Jaillant 2022; Jaillant and Rees 2023; Jaillant and Aske 2024a, 2024b; Jaillant et al 2025; Hutchinson 2020; Moss et al. 2018; Lee 2019). These authors have cited the unparalleled possibilities that AI technologies offer, particularly for facilitating classification and retrieval of vast amounts of historical records. Indeed, AI can be used to automatically create metadata, search vast amounts of historical records and answer questions with natural language, among many other applications. As the cultural heritage sector starts to embrace these novel technologies, it becomes imperative to examine their impact on diversity in collections.

Although Artificial Intelligence is becoming ubiquitous in other sectors, it is still at an early stage of development in libraries and archives. For example, the collection-as-data movement, which aims to highlight the potential of computational approaches applied to digital collections in the heritage sector, initially downplayed the importance of AI. The Santa Barbara Statement (Padilla et al 2019) did not even mention “Artificial Intelligence.” When it was superseded by the Vancouver Statement (Padilla et al 2023), AI appeared among the list of computational approaches, but was discussed mainly in terms of risks. “Collections as data may be used in training sets for Artificial Intelligence and product development,” declares the statement. “Stewards should be mindful of the ethical implications, including intellectual property and claims to data sovereignty, and should develop adequate safeguards against improper usage, detrimental loss of context, and the amplification of biases through these technologies.” Although these risks exist, there are also extraordinary opportunities to use AI to enhance the access and diversity of collections with AI.

Using a theoretical framework inspired by digital humanities and archival studies, this article is the first study to systematically explore the relationships between the lack of diversity of archival collections, and the application of AI in archives. In taking this untrodden path, our main argument is that the deployment of AI should involve close collaborations between developers of these technologies, archivists and librarians, particularly if the problem of lack of diversity in archives is to be properly addressed, and archives made more inclusive. Given the ongoing debates about AI and ethics, do AI and associated technologies compound or alleviate the lack of diversity in archives? Under what conditions can these technologies be most responsive to the need to diversify collections in archives and libraries?

The methodology of this article relies on expert insights from professionals drawn from government agencies, academia and some of the biggest cultural heritage organisations in the USA, UK and Europe (Norway, Sweden, Ireland, France, Spain). This focus on large-scale institutions is justified by the fact that few smaller archives and libraries have experimented with Artificial Intelligence, due in part to lack of funding and expertise. We paid attention to balance in gender (with ten women and ten men) and career stages (with a mix of early career and more senior professionals). Interviews took place in May and June 2024 via MS Teams, and lasted 35 min on average. All relevant ethical guidelines were followed in the conduct of this research. We obtained the full consent of interviewees to be named where appropriate in this article. Although our UK-based research team is relatively diverse (with researchers originally coming from Nigeria, the UK, France, and Spain), few of our interviewees are from the Global South. There are two reasons for this. First, since the focus of this article is on archives and libraries in the West, we did not approach professionals in other parts of the world. Second, the Archives and Libraries sector recruits mostly White professionals—and our interviewee sample reflects this lack of diversity in staffing.^7^ We nevertheless believe that the article’s insights will make an important contribution to current debates on diversity in archives, libraries and academia, debates that have largely neglected the role of technology in addressing (or reinforcing) historical imbalances in archival collections.

This article is organised in three main sections. After surveying the state of scholarship in the first part, we turn to the opportunities and risks associated with AI to address the lack of diversity in archival collections. The third section focuses on the need for closer synergies between those who develop AI systems and cultural heritage professionals. The conclusion includes practical recommendations that will help archivists, curators and other professionals to assess the opportunities and risks associated with AI and find solutions to make their collections more representative of diverse audiences.

## Literature review

Calls to diversify archival theory and practice have made a deep impact on the profession of archivist. The work of Michelle Caswell has been particularly influential. In a 2021 article, she called to shift “our thinking about the position of the archivist, from a purportedly objective ‘view from nowhere’ (which in fact belies a dominant but unnamed white male position), towards a socially located, culturally situated agent who centers ways of being and knowing from the margins” (Caswell 2021). Drawing on this call to embrace subjectivity and positionality, Jessica Tai (2023) has coined the concept of “cultural humility” to encourage archivists to pay more attention to their own biases and lack of neutrality. Tai argues that “an archival practice undertaken within a framework of cultural humility entails actively denouncing archival neutrality, requiring the continual and visible disclosure of own’s own positionality.” As a self-described “mixed-race, Chinese American cisgender woman who works as a processing archivist in a special collections library at an academic institution,” Tai claims that her own background is not irrelevant to her professional practice. On the contrary, it makes her well placed to design archival descriptions that are be less oppressive and discriminatory towards ethnic minorities.

In the archival sector, not everyone agrees that the archivist’s position should move from “objective” to more “subjective” and diverse. When it comes to the appraisal and selection of archival records, the subjectivity of archivists has traditionally been described as irrelevant at best, and even detrimental when it risks tempering with archival collections. In his book *Modern Archives: Principles & Techniques*, first published in 1956 and still influential today, T. R. Schellenberg writes: “The end of all archival effort is to preserve valuable records and make them available for use. Everything an archivist does is concentrated on this dual objective” (Schellenberg 1956). In this framework, there is no place for subjectivity: the value of records exists independently of archivists. Their role is to weed out records that are not valuable, and to ensure the integrity and authenticity of records, which are then preserved and made accessible.

Although archivists and other professionals do not all agree that a subjective positionality is needed to address imbalances and biases in their collections (Greene 2013), there is widespread agreement that these issues cannot simply be ignored: they need to be actively addressed. Data from marginalised or oppressed groups such as Indigenous and diasporic communities and people from formerly colonised territories exist; however, in many instances, they have not been brought to the forefront. Carter et al. (2006) argues that while archives contain lots of different voices, this does not mean that those voices are heard. A conscious acknowledgement of the gaps in archival narratives as well as the creation of new archives can address this issue, as well as past injustices.

This growing awareness of biases and silences in the archive draws on scholarship from a range of disciplines—including archival studies, digital humanities, museum studies, public history, memory studies, gender and ethnic studies. In a European context, diversity issues are rooted in colonial and imperial histories. Bastian (2006) points out that the narratives and voices of oppressed people are often neglected in archives. This is a result of the overwhelming “master narrative” that dominated colonial archives as the voice of the ruling elite. Chew (2023) articulates this further, arguing that practices within collections, including descriptive and cataloguing practices, “has served to privilege the perspectives and needs of white heterosexual cis-gender able-bodied men,” leading to silences of oppressed groups.

Growing voices, within and outside academia, have claimed that the patterns and infrastructures of power in place until the present postcolonial era have shaped societies and cultures from a place of inequality, exclusion and monocentric views. Decoloniality is articulated by Walsh and Mignolo (2018) as different processes and methods that undo and reject colonial dynamics and their consequences. As many libraries and archives have embraced the discourse of decolonisation when introducing EDI approaches to their operations and missions, a series of specific initiatives and methods have emerged. One approach is to consider the impact of coloniality in GLAMs and to call for reparative initiatives aimed at guaranteeing that equality, diversity and inclusion prevail in archives and museum documentation.

In the realm of cataloguing and documentation in GLAMs, technological tools are not viewed as neutral. Indeed, the recent decolonial turn in the digital humanities relies on the argument that digital tools, systems and platforms are devices shaped by colonial epistemologies that amplify both inequality and the lack of diversity that historically characterise collections datasets and narratives (Risam 2019). This argument is also explored in related fields such as critical data studies (Dalton et al. 2016) and decolonial computing and AI (Ali 2014; Adams 2021; Paraman and Anamalah 2023). The works of scholars that engage with this critical strand of the digital humanities envision possible pathways to unmask biases and embrace a diverse array of voices and narratives that would involve the use of digital tools for this purpose.

New media scholars are thus considering how the decolonial processes that reveal and reject the effects of colonialism could be implemented in a data-centric society defined by digital platforms and AI developments. Couldry and Mejias (2019) see datafication as a process related to mechanisms derived from colonialism such as extractivism and classification. They define “data colonialism” as practices that extract and capitalise data from ethnic minorities communities in Western countries and individuals from formerly colonised territories. The misuses of extracted data put individuals and communities at risk of being further controlled and discriminated. Bunz and Vrikki (2022) have developed the concept of “data solidarity” as a necessary element to be implemented when datasets are published and shared. Data solidarity implies the acknowledgement and increased visibility of the processes and biases behind the generation of datasets that could lead to harmful dynamics such as racism, sexism and classism. Self-reflection, criticality and acknowledgement are necessary to implement data solidarity in practice.

This distrust of technology is not shared by everyone. Richard Marciano and Victoria Lemieux, who have shaped the new discipline of computational archival science (CAS), deplore that “in recent archival discourse, technology has often been associated with colonialism and other negatively perceived ‘isms’” (Lemieux and Marciano 2024). For example, in 2011, the Pluralizing the Archival Curriculum Group (PACG) of the Archival Education and Research Institute declared that “archival studies education programs are conceptualized in strikingly similar ways worldwide, largely because of the overarching bureaucratically- and legally-centered paradigms developed and exported from Europe through colonialism, evangelism, mercantilism, and technological developments, and later codified through national and international standards and terminologies” (PACG 2011). For Lemieux and Marciano, technology is not at odds with the legitimate quest for equity and social justice. Pushing this point further, they argue that technological tools are essential to address these progressive themes, as demonstrated by the work of CAS scholars “focusing on ethical and social justice aspects of the intersection of computing and archival work” (Lemieux and Marciano 2024).

Technological tools such as AI can be very useful to address the misrepresentation of cultures and individuals in collections, as revealed by the language used in the documentation metadata. Focusing on historical photographs from the colonial period, the UK–French project EyCon uses computer vision to identify violent and otherwise problematic images and flag these materials with content warnings (Aske and Giardinetti 2023; Giardinetti et al 2024; Dentler et al. 2024). Many of these materials include captions and other metadata that rely on outdated language common during the colonial period. AI can be deployed to review this existing metadata, detect language that is racist or inappropriate and add new layers of metadata to provide new contextual elements and improve discoverability. Instead of having to use racist keywords to find materials, users can identify relevant pictures with more appropriate language. Likewise, projects such as Europeana’s DE-BIAS and Transforming Collections in the UK (Pui san lok et al. 2023)^8^ seek to tackle these language issues by training models that analyse vast numbers of records, primarily from museum collections, to help institutions detect and highlight the offensive and problematic terminology present in the documentation of objects. While AI can enhance efficiency and uncover new insights within archival records, the use of these technological tools also raises ethical questions regarding issues like data ownership, consent and cultural sensitivity. Since AI requires good quality data to be trained, there are debates about the perpetuation of bias, the replacement of human judgement and the maintenance of provenance.

The metaphor of fire has been applied to AI to encompass both the extraordinary potential of this technology and the huge risks associated with it. As Ben Buchanan and Andrew Imbrie show in *The New Fire,* Artificial Intelligence—if we manage it well—will become “a force for good” that “will be lighting the way to many transformative inventions” (Buchanan and Imbrie 2022). As traditional recordkeeping and documentation activities become less feasible with the growing volume of digital data, AI and associated technologies offer new hitherto unavailable methodologies to organise and access archival records. Scholars of AI applied to archives (Jaillant 2022; Colavizza 2021; Lee 2019; Hutchinson 2020) draw on this argument that computational methods can automate and process large datasets quickly, ensuring that valuable records are identified, preserved and made accessible. However, “if we deploy [AI] thoughtlessly, it will advance beyond our control” (Buchanan and Imbrie 2022). AI lacks the nuanced understanding required for accurate appraisal of archives. Human judgement is essential for contextual interpretation, ethical considerations and maintaining trust in the authenticity of records.

As evident from the scholarly literature, the integration of AI into archival practices presents both opportunities and challenges regarding diversity. While AI can enhance efficiency and uncover new insights within archival records, there are concerns about the lack of diversity, which in some instances is reinforced by AI and related technologies. We argue that a collaborative approach that leverages the strengths of both AI and human expertise from Archives and Libraries professionals is essential for addressing the lack of diversity and ensuring that archival practices remain ethical, inclusive and effective.

## AI and diversity: key issues, opportunities and risks

### Key issues

During our discussions, several interviewees mentioned the following three issues relating to the lack of diversity of archival collections. First, historical reasons explain the underrepresentation of records created by and/or representing people of colour, and the difficulties to access these collections. Second, the lack of diversity within Libraries and Archives professionals has an impact on decision-making and on the prioritisation of certain records over others. The first two issues feed into a third one: the issue with ownership and interpretations of archival collections. In particular, the metadata used to describe colonial collections is increasingly contested when it includes racist language and otherwise problematic terms.

The idea that libraries and museums should be accessible to a wide audience, rather than a small elite, can be traced back to the Enlightenment. The French Revolution opened up national archives to all citizens by instituting a 1794 law that created “a central repository for the national archives,” with free public access (Favier 2004). But the idea of representing diverse audiences in collections is a much more recent construct. It coincides with demands for greater representativeness in other fields and disciplines, such as literature. As John Guillory (1993) notes, the use of the term “canon” in literary studies is relatively recent. Until the 1970s, ‘it was still possible to discuss what we call canon formation exclusively by reference to the word ‘classic’” The “canon wars” of the 1990s opposed, on the one side, those who described literary reputation as a historical construction dependent on the social, political and commercial interests of the time, and on the other side, those who continued to follow the more traditional approach epitomised by the nineteenth century writer Matthew Arnold who famously viewed the canon as “the best that has been thought and said.” For proponents of the first approach, the literary canon—which had so far been dominated by White males—had to become more diverse to include more women and Black writers in particular.

Like literature professors, archivists and curators have inherited an earlier situation where representativeness was not a concern. As Benjamin Lee (Assistant Professor at the University of Washington’s Information School) argues, lack of diversity emanates from, and occurs throughout “a genealogy of work and cultural heritage where the issues go back to fundamental challenges or lack of collecting that was done 100 or 200 years ago.”^9^ This perspective is also echoed by Jean-Philippe Moreux (AI Scientific Advisor at the Bibliothèque Nationale de France) who views the issue of lack of diversity as “driven by historical events” evidenced in legal deposit collections at the BNF.^10^ History therefore plays a significant role in underrepresentation as witnessed in present-day collections.

Many libraries, archives and museums recognise the need to improve the inclusivity and representativeness of their collections. Arran Rees (Postdoctoral Research Fellow on the Congruence Engine project)^11^ thus declared: “the GLAM sector is largely white and middle class. And although it has quite an even spread of gender, the collections remain largely white, cis- hetero, able bodied and patriarchal. So that’s both in the objects themselves, but also very much so in the metadata.”^12^ This is reflected in the decision-making processes within cultural heritage institutions. As Laura Gibson (Lecturer/Assistant Professor in Digital Content at King’s College London) argues: “There’s a lack of diversity in terms of who gets to make those decisions, about what’s important and what needs to be recorded.”^13^ The ways that collections have been catalogued, stored, presented and over/underprioritised demonstrate that long-standing diversity issues within the GLAM sector are not only historic, but still have significant ramifications today.

Ownership and interpretations of archival collections is a key issue for many cultural heritage institutions. Referring to her work on early Colonial Mexico, Patricia Murrieta-Flores (Professor of Digital Humanities at Lancaster University, UK) argues: “These are live documents, so this is to say that many communities, for instance, are owners of these documents. They held these documents, and they are complex.”^14^ The beliefs and traditions of the original creators of records have sometimes been marginalised or even erased from the archive. For example, specific items in collections may be interpreted in different ways by different groups, but metadata may not have this diversity attached to it.^15^ This is a problem across the GLAM sector, in archives but also in museums. Laura Gibson recalled that when she worked with the Iziko South African Museum, one of their visitors was a Sangoma (a traditional healer) who came to see an object described as a medicine container in the catalogue. “He wasn’t interested in it being that; he was interested in the spiritual element that was in there, and there wasn’t anywhere, of course, on the catalogue card to put that because it requires a whole reorganisation and assessment of belief.”^16^ Diversifying catalogue descriptions and adding new metadata (when racist and problematic terms are used in the original metadata) are therefore the central objectives.

### Using AI to diversify archival collections

As Simon Popple (Academic Lead for the Digital, Creativity and Cultures Hub at University of Leeds) pointed out, Artificial Intelligence can be used to speed up the creation of descriptions at catalogue level. It can also accelerate processes with linking data, tagging and cross-referencing. “It’s got the opportunity to really revolutionise the role of the curator to speed things up,” adds Popple.^17^ In the Libraries and Archives sector, which values the stability necessary to preserve historical records over the long term, the rapid pace of AI is largely seen as disrupting. In his report on AI for libraries, Ryan Cordell strongly criticises the Silicon Valley ideology epitomised by Facebook’s former motto, Move fast and break things. He insists that libraries and scholars should focus on building, not breaking. They should move slowly and deliberately, turning their backs on the unethical practices of tech giants. At the same time, libraries should “not wait for the data to be perfect, but instead present it as a pilot or prototype, learn from users, and refine from there” (Cordell 2020). This ambiguity towards technological progress and speed was shared by several of our interviewees, who praised the opportunities offered by AI, but were also worried by the disruptions it would bring.

Since cataloguing is a time-consuming and expensive process, AI is presented as a cost-saving measure to diversify metadata at scale. Indeed, relying only on archivists, librarians and volunteers from source communities is not practical or even ethical. As Laura Gibson pointed out, people who are asked to contribute their knowledge to diversify collections, for example via crowdsourcing, are often “not being compensated for that in a fair and equitable way for their labour.”^18^AI cannot replace humans, but it can help create new layers of metadata at scale and for a low cost. Likewise, Jenny Bunn (Head of Cataloguing, Taxonomy and Data, The National Archives UK) pointed out the usefulness of AI-powered tools such as video summarisation, caption generation and handwritten text recognition. “They reduce the amount of work that we have to do and they also provide something that might allow us to have some kind of control over this ever-growing digital stuff.”^19^

AI is a necessity to address the exponential growth of digital records, with additional demands on archivists’ time brought by remediation of formats and demands for diversifying metadata. Jenny Bunn said:

What people think of as the data that they want to work with—and this is perhaps from a research context—is growing. It used to be a piece of paper. Then it was an image on screen. Now it’s a structured database probably expressed in JSON that can just be sucked into an AI pipeline. There’s work involved in all of their transformations, and quite often that falls on the archive.

For Bunn, the heavy workload and pressure on archivists are unsustainable and need to be addressed not only with AI, but also with deaccessioning (i.e. the process by which an item is permanently removed from a cultural heritage collection). “There certainly aren’t limitless resources going into cultural heritage at the moment,” Bunn declared. Keeping the work to a manageable amount is essential to ensure the sustainability of the archive sector.

The automatic creation of metadata—especially on sensitive archival records—cannot be done without inputs from humans. Likewise, enriching language models requires the participation of various communities. Javier de la Rosa (Senior Research Scientist at the National Library of Norway’s AI lab) mentioned their collaborative work with the Sami population, the only indigenous population left in Europe. Three different communities of Sami people live in Norway, with their own languages and culture. These languages have traditionally been neglected, which has an impact on the digital tools such as OCR used by the library to transcribe books:

When we started scanning, … the tools were OK-ish for Norwegian, but when we scanned books written in their languages, it was just transcribed as garbage. It was not possible to read the actual text written in other languages. So even if we do not intend to impose any bias, the technological bias, that is still there and it’s not easy to fix or tackle.^20^

In January 2024, the National Library of Norway published an automatic speech recognition model for the Norwegian languages. “Sami was not part of that,” de la Rosa said, “but we collaborated with a university in the north in Tromsø and with the Sami community. So they provided us with some annotated samples of the speech in the Sami language, and now we are integrating that into our official models so they feel that they are also represented and served.” A combination of insights from humans and automation can therefore be used to address the need to diversify languages in archival collections.

AI is increasingly applied to archival images, in addition to text, to improve the discoverability of hidden collections in particular. Jeff Steward (Director of Digital Infrastructure and Emerging Technology at Harvard Art Museums) mentioned that about ten years ago, he started exploring the use of computer vision as a tool for building up additional descriptive data about neglected collections. The central objective was to enhance discoverability and access to these little-known items. However, greater access is not always desirable, for example in the case of contentious and culturally insensitive materials. Steward said:

Every once in a while something will bubble up, like a curator will find something in our collection that they’ve just never seen before and they’re like, “Oh, we really shouldn’t have that online. We should remove that. Or we should have a click-through warning or something like that.”

… the AI could help us do that at scale, like in a couple of days we could run the whole collection through a couple of times and at least start to understand the universe of our collection enough to find the corners of the collection we really need to look at and address.^21^

In very large collections, manually reviewing each item is not feasible, and AI can therefore be of great help to spot problematic materials.

### Risks associated with AI

While the application of AI technologies in archives and libraries opens up hitherto unimaginable possibilities, it also comes with a degree of potential pitfalls. Several of our interviewees thus mentioned the black box problem. Clemens Neudecker (Researcher at Berlin State Library) noted that “there is a big risk in the sense that many of these AI technologies, especially from industry, [are] like a black box. So, you cannot really look into what drives the system, what are the, again, biases or issues that that technology has in itself like how it has been produced.”^22^ Neudecker added that he and his colleagues do not use ChatGPT because of its lack of transparency about the sources that were used to train the AI. Benjamin Lee (University of Washington) echoed the same concerns when he stated that “I think there are a lot of questions around data privacy, especially when we apply AI tools, where we’re relying on some sort of opaque algorithm or something that’s proprietary in some way.”^23^ The lack of understanding in the decision-making process runs against key principles in the Libraries and Archives sector, such as the needs for transparency and open access. Neudecker pointed out that the Berlin State Library is developing its own AI tools and models, provided open source to offer “as much control and transparency as possible.”^24^

In addition to the back box issue, AI is also associated with the risk of replicating biases in archival collections. As Benjamin Lee put it, “large language models are training on massive amounts of cultural heritage data.”^25^ This has an impact on the responses produced by generative AI tools, which may replicate diversity problems already embedded in collections. Marlene Daut (Professor of French and African Diaspora Studies, Yale University) has spoken about this issue.^26^ She works on Haiti and began digitising local periodicals and making them accessible online (https://lagazetteroyale.com/). She has also done work collecting and digitising images of the Haitian revolution, contextualising them, making them available (see https://www.haitianrevolutionaryfictions.com/). Historical context is important because much of the images and texts represent the perspective of the colonisers. But as she made this material available online, she realised that it then became fodder for large language models which will machine read, disconnected from historical context. The risk is one of perpetuating the myth of the colonial mind.

Javier de la Rosa (National Library of Norway) gave us an example of how issues relating to the lack of diversity in collections—such as the prejudices against Indigenous people—can feed into AI systems.

There are communities that have been mistreated in the history of the country, so when we trained these models, we fed them everything that was in the newspapers of the last century. We have encoded all that hate speech into the model. If you look for things like ugly people, some images might pop up that are very offensive to specific communities… We do have a huge responsibility when we release these models. These are not neutral artefacts in any way, they are biased machines. They are designed to exploit the patterns in the text and we really need to be careful with that.^27^

This call to design responsible AI systems, trained on a wide range of data, is echoed by other interviewees. Simon Popple (University of Leeds) stated: “I think the greatest problem is the fact that the data that the AIs are trained on is partial. It’s from the Global North. It ignores most cultures so it’s very problematic. And so, the representations or misrepresentations that are possible are really quite ethically and morally very, very dangerous.”^28^ Ultimately, applying AI to archival materials—especially sensitive materials—is fraught with ethical difficulties. This was well articulated by Benjamin Lee who describes the ethical practices when cataloguing archives relating to Holocaust victims and survivors, a subject that should be dealt with upmost sensitivity. He asked: “What does it mean to datafy individuals and, for example, to provide unique identifiers, or numbers to people who’re part of the dehumanizing experience of the camp?”^29^ Issues such as lack of consent of data “subjects” can be duplicated when AI is applied to sensitive collections.

Professional guidelines for dealing with sensitive records in the AI age are therefore essential. For collections that contain materials impacted by colonial legacies, GLAM organisations already have guidelines on, for example, consulting representatives from communities whose cultures and histories may be catalogued or displayed. This is particularly relevant in discussions as to whether objects should be in collections, or objects should be removed (Bursey 2022) and physically or digitally repatriated (Bell et al 2013). Clemens Neudecker cited ongoing self-regulation initiatives that have been set up by the GLAM organisations at the European level:

Together with a network of European GLAM organisations in the frame of Europeana, the European digital library,… we’re creating a template to describe cultural heritage data that is being put online with a particular emphasis on documenting issues such as diversity, racism, etc.,… for others to reuse to drive adoption of that.^30^

But these guidelines do not fully take into account the opportunities and risks that come with AI applied to contested collections.

It is one thing to design guidelines within the GLAM sector, and it is another to push for national and international regulatory frameworks to guide the use of AI. These frameworks would, in Ida Varošanec’s (2022) words, “contain clear transparency obligations so that impacted individuals as well as innovators are empowered to use and trust AI systems”. However, many interviewees were unsure how this would be implemented in the GLAM sector alone. Simon Popple articulated the need for having sector-specific guidelines rather than broader regulations: “for the sector itself, it’s something that should be self-determined and self-organised rather than having something imposed which I think it inevitably might happen.”^31^ There seems to be a sense that the GLAM sector, at least in Europe, wants to maintain its freedom in determining its own regulatory parameters. While there is an expectation that AI should be used in a way that is most beneficial for GLAM specifically, progress is hindered by the lack of collaboration between AI specialists and GLAM sector professionals.

## Fostering more collaborations between AI specialists, archivists and librarians

Although there have been attempts to make AI more responsive to diversity issues, such attempts have rarely involved archivists and librarians, and have in some cases reinforced the problem. As Nicole Coleman (Digital Research Architect for Stanford University Libraries) noted,

a machine learning system is inherently limited and static. As a result, tech companies have to come up with engineered fixes on top of the model in an attempt to make the outputs “diverse.”… With Google’s Gemini, for example, they recently released a new model which included a diversity prompt that was hidden from the person using the chatbot. It would make whatever question anyone asked more “diverse,” whatever that means. They were trying to solve the problem of lack of diversity. But their solution is absurd because lack of diversity is built into the system.^32^

To address diversity, Gemini thus generated images of Black Nazi soldiers, thereby disregarding historical realities as well as racial sensitivities. For Coleman, the real problem is that Google and other tech companies release these tools to the public, without explaining to users what the limitations are. “People think they’re getting truth or facts,” rather than AI-generated hallucinations.

Nicole Coleman gave another example of problematic large language models (LLMs) to highlight the consequences of handing over responsibility for important decisions to the technologies and those who are developing them. One of Coleman’s colleagues was introduced to the possibilities of using Google’s Gemini to transcribe images of handwritten letters. They used the tool on the correspondence of an American diplomat in the Dominican Republic in the late nineteenth century. The letters contain conversations about slavery. Coleman’s colleague discovered that “the model simply shuts down when it comes across text that the safety settings determine to be harmful in some way. It just simply will not fulfil the transcription task.” For Coleman, this is particularly worrying, since it transfers the control from the human researcher to the AI tool.

This is what I think is very concerning about the notion of designing AI safety for these kinds of models. The intention may be good, but the implementation is devoid of theory. In their attempt to protect us from ourselves, the engineering decisions effectively remake our understanding of content, potentially of the past.

Here, Coleman pointed out the long-term consequences of this loss of control: the fact that AI tools have the potential to influence the way we do research and the way we write history.

Closer collaboration between those who design AI systems and GLAM sector professionals is hindered by mistrust and lack of enthusiasm for tools designed by tech giants. Giles Bergel (Senior Researcher in Digital Humanities, University of Oxford) mentioned that the AI debate should be led by “archives and libraries and museums, whose duty it is, whose mission it is to curate not just an object, but the understanding of that object and help with interpretations of it.” He pointed out that at the AI safety summit organised at Bletchley Park in 2023, the GLAM sector was not represented. “There were, to be fair, some prominent AI ethics people” and “programmes like BRAID [on responsible AI] are doing great work in this regard. But really the [GLAM] sector should be leading on this,” Bergel argued. And he added: “I worry a bit about the AI risk and fairness debate being outsourced to professionals who aren’t embedded in the domain.”^33^

As was the case with digitisation, many archivists and librarians see AI as a necessity that they must adopt, rather than a great opportunity to enhance collections and reach new audiences. As W. J. von Eschenbach (2021) argues, “our relationship with technology often is one of reliance rather than trust.” Furthermore, several interviewees articulated the difficulty in educating people on AI literacy with limited resources.^34^ Nicole Coleman observes that “the biggest challenge that we face is the insufficient investment in libraries, archives, and museums, to apply this technology as they choose to do.”^35^ Pushing this point further, Javier de la Rosa argued that the relative lack of resources in the Libraries and Archives sector leads to an overreliance on existing AI models which are promoted for profit. He stated that tech companies “do not care for the same issues we care, and they are not going to tailor models for our own purposes.”^36^ Here, de la Rosa’s “they versus us” discourse highlights the wide gap between AI companies and the cultural heritage sector.

There is also a sense that the limited involvement of librarians and archivists in the development of AI leaves the sector exposed to the control of the tech industry. Michael Ridley (Librarian Emeritus at the University of Guelph) argued: “If we don’t get involved, we’re going to be beholden to other forces that will decide these things for us.”^37^ AI should be used in a tailored way that is most beneficial for the cultural heritage sector, argued Giulia Osti (PhD Candidate in AI and Preservation at University College Dublin). “[AI] doesn’t really seem to be fitting our needs that much.”^38^ To bridge the gap between experts in tech companies and GLAMs, Daniel van Strien et al (2021) have proposed an introductory machine learning training, “that is grounded in the specific applications and use cases relevant to cultural heritage, that is practical, without being too overtly technical.” This training “will be key to ensuring the wider adoption of ML methods across GLAM.” Without dealing with this issue, it will be difficult to address further problems regarding diversity and bias.

A collaborative approach to the development and application of AI between archivists, librarians and AI experts could lead to productive new ways to tackle diversity and bias problems. Nicole Coleman pointed out the dangers of using closed systems, where tools must be used “either on a particular dataset that the vendor is providing, or just to perform a specific technical task. Closed systems fragment the work of information specialists.” For Coleman, “power tools for librarians” are needed to offer more control to cultural heritage professionals:

If we instead educate our librarians about the potential of this technology and then bring in technologists to collaborate with librarians, we can do tremendous things. The limitations in terms of diversity of materials, will be the limitations that already exist. In other words, the ongoing work to diversify collections will be a social and organisational problem rather than a technical constraint. The technology will be put to use to support the work is being done, well or poorly, by human beings.

According to Coleman, the “technology has to be directed by the librarians themselves, not by the manufacturers of the tools.”^39^

The need to take back control over technology is all the more important that archivists, librarians and humanities scholars bring unique perspectives that have so far been largely ignored. Nicole Coleman reminded us that librarians are trained to understand and make choices “about curation, selection, assessment.” This expertise in data and record management is extremely valuable, for example to prepare datasets used to train AI systems. Likewise, humanities scholars bring essential input on ethics, long-term historical perspectives, and critical thinking on technology and AI. This was articulated by Patricia Murrieta-Flores (Lancaster University), who said:

The humanities, in terms of philosophy, can already tell you why a fully automatic system is not a good idea. Historians can point you to all those points in time where disruptive technologies were dreadful for humanity. Literary scholars have basically explored through creative enterprise and writing with sci-fi the many different scenarios that could happen if something goes wrong. There is quite a lot, for instance, in the humanities that we have thought critically.^40^

Humanistic values and perspectives, which prioritise the well-being, dignity and equality of all individuals could mitigate some of the risks associated with AI. As Nicole Coleman argued, we need to bridge the “chasm between engineering technology and humanistic values.” One way of doing that to foster closer collaborations and “put the tools in the hands of individuals who are trained with those values.”^41^ This call to take back control over technology and to embed humanistic values adds to current movements such as *AI for Good*, or the work of scholars who see AI and technologies as tools for societal change that can contribute to greater diversity, inclusion and equality (Gallon 2016).

## Conclusion

In his 1994 article “Electronic Records, Paper Minds,” Terry Cook wrote about the emergence of a new digital world replacing the old paper-based world, and the impact that this upcoming revolution would have on archivists and record managers (Cook 1994). “If we as information professionals can guide our sponsors and users from masses of specific information on to knowledge, and even wisdom, we will be secure indeed in the new age and make a valuable contribution to society and posterity,” Cook argued. “If not, we will be replaced by software packages that can handle facts, and data, and information very efficiently, without any mediation by archivists or anyone else.” Thirty years later, archivists and records professionals have not disappeared, but their expertise on data has been marginalised in a sector dominated by tech giants. Many of our interviewees felt that they lacked power, and that it was time to take back control by co-designing “power tools” that would put the needs of the Libraries and Archives sector first. These new tools would be especially useful to address issues with the diversity of archival collections.

Indeed, the lack of collaboration between archivists, librarians and AI developers is one of the most significant impediments to the ethical and inclusive application of AI in collections—especially in collections that contain sensitive historical materials. While AI tools offer unprecedented opportunities for huge archival collections, they currently lack the nuances and sophistication required for accurate appraisal and cataloguing of these collections. Human judgement and oversight are essential for contextual interpretation, ethical considerations and maintaining the provenance and authenticity of records. Bridging the gap between creators of AI technologies and cultural heritage professionals is a necessary step to take back control and make sure that technology works for humans, and not the other way around.

Such collaboration can take many forms, and we would like to make the following three recommendations.

- Investing in interdisciplinary training programmes is essential to equip archivists with the necessary skills to leverage AI technologies effectively.

The discipline of Computational Archival Science emerged as a response to this need for closer engagement between archivists and technology experts. But it is still in the early stages of development and suffers from criticism that associates technology with colonialism and Western domination. It is important to recognise that technology and social justice are compatible, and AI can be a powerful tool to address issues with diversity in archival collections. For archivists, having a good understanding of AI is a necessary step to engage with tech experts and shape the design of tools.

- Similarly, educating AI developers about the unique needs and challenges of archival data management can foster more relevant solutions.

Too often, AI tools are designed without input from archivists and knowledge and information professionals. The motto “Move fast and break things” that once dominated the Silicon Valley left little time for consultation outside narrow groups of tech specialists. But this lack of collaboration can lead to problematic outcomes, such as the reinforcement of existing biases in a collection.

- Professional guidelines are needed to determine the best way to use AI to address the lack of diversity in archival collections.

Too often, efforts to diversify collections (for example by adding new layers of metadata) are done in silo within a specific library or archival institution. As previously described, initiatives exist to provide sector-wide guidance—such as the templates prepared by the European digital library Europeana, which document issues such as racism, and are designed to be reused by other institutions. But there is a current lack of guidelines on the specific issues brought by AI applied to archives, including the risk of replicating existing biases. There is an urgent need to work closely with AI professionals to co-design professional guidelines that would frame the application of ethical and responsible AI to address the critical issue of lack of diversity in archival collections.

## Data Availability

No datasets were generated or analysed during the current study.

## Footnotes

1 The Oxford English Dictionary defines "diversity" in various contexts, including its nuanced meaning in Sense 1.d. For more details, see: Oxford English Dictionary, Oxford UP, July 2023, https://doi.org/10.1093/OED/3622867053.

2 Galleries, Libraries, Archives and Museums.

3 Science Museum Group, https://learning.sciencemuseumgroup.org.uk/our-approach/an-equitable-approach-to-engaging-people-with-science/ Accessed 6 November 2024.

4 Art Institute of Chicago, https://www.artic.edu/about-us/identity-and-history/equity Accessed 7 November 2024.

5 Gabriele Griffin, interview via MS Teams, 30 May 2024.

6 Te Mana Raraunga, 2022. Māori Data Sovereignty Network. https://www.temanararaunga.maori.nz Accessed 19 November 2024.

7 In the UK, a 2015 study of the information workforce by ARA (Archives and Records Association) and the Chartered Institute of Library and Information Professionals (CILIP) found that 97.7% of the archives workforce identify as White; this is in comparison to 87.5% of the overall UK workforce (ARA and CILIP 2015). In the USA, a 2017 survey conducted by the Women Archivists Section garnered 2170 responses (including both SAA [Society of American Archivists] members and non-members). This sample was 87.7% White, 3.1% African American, 3.6% Latinx, and 3.2% biracial or multiracial (Israel and Eyre 2017).

8 The Transforming Collections project partly expands on “Provisional Semantics,” mentioned earlier in this article.

9 Benjamin Lee, interview via MS Teams, 29 May 2024.

10 Jean-Philippe Moreux, interview via MS Teams, 5 June 2024.

11 https://www.sciencemuseumgroup.org.uk/projects/the-congruence-engine Accessed 21 November 2024.

12 Arran Rees, interview via MS Teams, 24 May 2024.

13 Laura Gibson, interview via MS Teams, 24 May 2024.

14 Patricia Murrieta-Flores, interview via MS Teams, 29 May 2024.

15 Laura Gibson, interview via MS Teams, 24 May 2024.

16 Laura Gibson, interview via MS Teams, 24 May 2024.

17 Simon Popple, interview via MS Teams, 7 June 2024.

18 Laura Gibson, interview via MS Teams, 24 May 2024.

19 Jenny Bunn, interview via MS Teams, 24 May 2024.

20 Javier de la Rosa, interview via MS Teams, 28 May 2024.

21 Jeff Steward, interview via MS Teams, 7 June 2024.

22 Clemens Neudecker, interview via MS Teams, 22 May 2024.

23 Benjamin Lee, interview via MS Teams, 29 May 2024.

24 Clemens Neudecker, interview via MS Teams, 22 May 2024.

25 Benjamin Lee, interview via MS Teams, 29 May 2024.

26 Example given by Nicole Coleman, interview via MS Teams, 29 May 2024.

27 Javier de la Rosa, interview via MS Teams, 28 May 2024.

28 Simon Popple, interview via MS Teams, 7 June 2024.

29 Benjamin Lee, interview via MS Teams, 29 May 2024.

30 Clemens Neudecker, interview via MS Teams, 22 May 2024.

31 Simon Popple, interview via MS Teams, 7 June 2024.

32 Nicole Coleman, interview via MS Teams, 29 May 2024.

33 Giles Bergel, interview via MS Teams, 29 May 2024.

34 Laura Gibson, interview via MS Teams, 24 May 2024; Arran Rees, interview via MS Teams, 24 May 2024; Jenny Bunn, interview via MS Teams, 24 May 2024; Simon Popple, interview via MS Teams, 7 June 2024.

35 Nicole Coleman, interview via MS Teams, 29 May 2024.

36 Javier de la Rosa, interview via MS Teams, 28 May 2024.

37 Michael Ridley, interview via MS Teams, 3 June 2024.

38 Giulia Osti, interview via MS Teams, 28 May 2024.

39 Nicole Coleman, interview via MS Teams, 29 May 2024.

40 Patricia Murrieta-Flores, interview via MS Teams, 29 May 2024.

41 Nicole Coleman, interview via MS Teams, 29 May 2024.

## References

[CR1] Adams R (2021) Can artificial intelligence be decolonized? Interdiscip Sci Rev. 10.1080/03080188.2020.1840225

[CR2] Ali M (2014) Towards a decolonial computing. Ambiguous technologies: philosophical issues. Human Nature, International Society of Ethics and Information Technology, Practical Solutions, pp 28–35

[CR3] ARA and CILIP (2015) A study of the UK information workforce. Mapping the library, archives, records, information management and knowledge management and related professions in the United Kingdom. http://www.archives.org.uk/images/Workforce/A_study_of_the_information_workforce_141115_7mb.pdf

[CR4] Aske K, Giardinetti M (2023) (Mis)Matching metadata: improving accessibility in digital visual archives through the EyCon project. J Comput Cult Herit. 10.1145/3594726

[CR5] Bastian J (2006) Reading colonial records through an archival lens: the provenance of place, space and creation. Arch Sci 6:267–284. 10.1007/s10502-006-9019-1

[CR6] Bell J et al (2013) After the return: digital repatriation and the circulation of indigenous knowledge workshop report. Museum Worlds 1:195–203. 10.3167/armw.2013.010112

[CR7] Bingham N, Byrne H (2021) Archival strategies for contemporary collecting in a world of big data: challenges and opportunities with curating the UK web archive. Big Data Soc. 10.1177/2053951721990409

[CR8] Buchanan B, Imbrie A (2022) The new fire: war, peace, and democracy in the age of AI. MIT Press, Cambridge, Massachusetts

[CR9] Bunz M, Vrikki P (2022) From big to democratic data: why the rise of ai needs data solidarity. In: Filimowicz M (ed) Democratic frontiers: algorithms and society. Routledge, London/New York, pp 47–62

[CR10] Bursey L (2022) Colonial-looted cultural objects in England. Santander Art Cult Law Rev 8:341–354. 10.4467/2450050XSNR.22.031.17044

[CR11] Burton AM (2005) Introduction: archive fever, archive stories. In: Burton AM (ed) Archive stories: facts, fictions, and the writing of history. Duke University Press, Durham, NC, pp 1–24

[CR12] Carroll SR et al (2020) The CARE principles for indigenous data governance. Data Sci J. 10.5334/dsj-2020-043

[CR13] Carter R (2006) Of things said and unsaid: power, archival silences, and power in silence. Archivaria 61:216–233. https://archivaria.ca/index.php/archivaria/article/view/12541

[CR14] Caswell M (2021) Dusting for fingerprints: introducing feminist standpoint appraisal. J Crit Library Inf Stud. 10.24242/jclis.v3i2.113

[CR15] Chew C (2023) Decolonising description: addressing discriminatory language in Scottish public heritage and beyond. J Irish Scot Stud. 10.57132/jiss.213

[CR16] Chilcott A (2019) Towards protocols for describing racially offensive language in UK public archives. Arch Sci 19:359–376. 10.1007/s10502-019-09314-y

[CR17] Colavizza G et al (2021) Archives and AI: an overview of current debates and future perspectives. J Comput Cult Herit 15(1):1–15. 10.1145/3479010

[CR18] Cook T (1994) Electronic records, paper minds: the revolution in information management and archives in the post-custodial and post-modernist era. Archives and Manuscripts 22:300–328. https://publications.archivists.org.au/index.php/asa/article/view/8433. Accessed 21 Nov 2024

[CR19] Cordell R (2020) Machine learning + libraries. Library of Congress, Washington, D.C. https://labs.loc.gov/static/labs/work/reports/Cordell-LOC-ML-report.pdf?loclr=blogsig. Accessed 21 Nov 2024

[CR20] Couldry N, Mejias UA (2019) Data colonialism: rethinking big data’s relation to the contemporary subject. Telev New Media 20(4):336–349. 10.1177/1527476418796632

[CR21] Dalton CM, Taylor L, Thatcher J (2016) Critical Data Studies: A dialog on data and space. Big Data Soc. 10.1177/2053951716648346

[CR22] Dentler J, Jaillant L, Foliard D, Schuh J (2024) Sensitivity and access: unlocking the colonial visual archive with machine learning. Digit Humanit Q 18. https://www.digitalhumanities.org/dhq/vol/18/2/000742/000742.html

[CR23] Favier L (2004) La mémoire de l’État: histoire des archives nationales. Fayard, Paris

[CR24] Gallon K (2016) Making a case for the black digital humanities. In Gold MK, Klein LF (eds) Debates in the Digital Humanities 2016. University of Minnesota Press, Minneapolis. https://dhdebates.gc.cuny.edu/read/untitled/section/fa10e2e1-0c3d-4519-a958-d823aac989eb

[CR25] Giardinetti M et al (2024) The EyCon dataset: a visual corpus of early conflict photography. J Open Humanit Data. 10.5334/johd.213

[CR26] Greene M (2013) A critique of social justice as an archival imperative: what is it we’re doing that’s all that important? Am Archivist 76:302–334. 10.17723/aarc.76.2.14744l214663kw43

[CR27] Guillory J (1993) Cultural capital: the problem of literary canon formation. University of Chicago Press, Chicago

[CR28] Harrison R et al (2020) Heritage futures: comparative approaches to natural and cultural heritage practices. UCL Press, London

[CR29] Hutchinson T (2020) Natural language processing and machine learning as practical toolsets for archival processing. Rec Manag J 30(2):155–174. 10.1108/RMJ-09-2019-0055

[CR30] Israel RH, Eyre JR (2017) The 2017 WArS/SAA salary survey: initial results and analysis. Women Archivists Section. Society of American Archivists. https://www2.archivists.org/sites/all/files/WArS-SAA-Salary-Survey-Report.pdf

[CR31] Jaillant L (2022) Archives, access and artificial intelligence: working with born-digital and digitized archival collections. Transcript, Bielefeld, Germany

[CR32] Jaillant L, Aske K (2024b) Are users of digital archives ready for the AI era? Obstacles to the application of computational research methods and new opportunities. J Comput Cult Herit 16(4):1–16. 10.1145/3631125

[CR33] Jaillant L, Rees A (2023) Applying AI to digital archives: trust, collaboration and shared professional ethics. Digit Scholarsh Humanit 38(2):571–585. 10.1093/llc/fqac073

[CR34] Jaillant L, Warwick C, Gooding P, Aske K, Layne-Worthey G, Downie JS (eds) (2025) Navigating artificial intelligence for cultural heritage organisations. UCL Press, London

[CR35] Jaillant L, Aske K (2024a) AI and medical images: addressing ethical challenges to provide responsible access to historical medical illustrations. Digit Humanit Q 18. https://www.digitalhumanities.org/dhq/vol/18/2/000755/000755.html

[CR36] Lee B (2019) Machine learning, template matching, and the international tracing service digital archive: automating the retrieval of death certificate reference cards from 40 million document scans. Digit Scholarsh Humanit 34(3):513–535. 10.1093/llc/fqy063

[CR37] Lemieux VL, Marciano R (2024) Teaching computational archival science: context, pedagogy, and future directions. https://ai-collaboratory.net/wp-content/uploads/2024/09/Teaching-Computational-Archival-Science-Context-Pedagogy-and-Future-Directions-2024-submitted.pdf

[CR38] Moss M et al (2018) The reconfiguration of the archive as data to be mined. Archivaria 86:118–51. https://archivaria.ca/index.php/archivaria/article/view/13646

[CR39] PACG (Pluralizing the Archival Curriculum Group) (2011) Educating for the archival multiverse. Am Archivist. 10.17723/aarc.74.1.hv339647l2745684

[CR40] Padilla T et al (2019) Santa Barbara statement on collections as data—always already computational: collections as data. Zenodo. 10.5281/zenodo.3066209

[CR41] Padilla T et al (2023) Vancouver statement on collections as data. Zenodo. 10.5281/zenodo.8342171

[CR42] Paraman P, Anamalah S (2023) Ethical artificial intelligence framework for a good AI society: principles, opportunities and perils. AI Soc 38(2):595–611. 10.1007/s00146-022-01458-3

[CR43] Pringle E, Mavin H, Greenhalgh T, Dalal-Clayton A, Rutherford A, Bramwell J, Blackford K, Balukiewicz K (2022) Provisional semantics: addressing the challenges of representing multiple perspectives within an evolving digitised national collection. Zenodo. 10.5281/zenodo.7081347

[CR44] Pui San Lok S et al (2023) Second report—transforming collections: reimagining art, nation and heritage. 10.5281/zenodo.7967237

[CR45] Risam R (2019) New digital worlds: postcolonial digital humanities in theory, praxis, and pedagogy. Northwestern University Press, Evanston

[CR46] Schellenberg TR (1956) Modern archives: principles and techniques. University of Chicago Press, Chicago

[CR47] Stock K (2020) Material girls: why reality matters for feminism. Hachette, London

[CR48] Stoler AL (2008) Along the archival grain: epistemic anxieties and colonial common sense. Princeton University Press, Princeton, NJ

[CR49] Van Strien D et al (2021) An introduction to AI for GLAM. In: Proceedings of the 2^nd^ teaching in machine learning workshop. https://proceedings.mlr.press/v170/strien22a/strien22a.pdf

[CR50] Tai J (2023) Cultural humility as a framework for anti-oppressive archival description. In: Anderson G (ed) Reinventing the museum: relevance, inclusion, and global responsibilities. Rowman & Littlefield, Lanham, Maryland, pp 349–364

[CR51] Varošanec I (2022) On the path to the future: mapping the notion of transparency in the EU regulatory framework for AI. Int Rev Law Comput Technol 36(2):95–117. 10.1080/13600869.2022.2060471

[CR52] von Eschenbach WJ (2021) Transparency and the black box problem: why we do not trust AI. Philos Technol 34(4):1607–1622. 10.1007/s13347-021-00477-0

[CR53] Walsh CE, Mignolo W (2018) On decoloniality: concepts, analytics, practices. Duke University Press, Durham/London

